# Acute dyspnoea and single tracheal localisation of mantle cell lymphoma

**DOI:** 10.1186/1756-8722-3-34

**Published:** 2010-09-28

**Authors:** Jean-Christophe Ianotto, Adrian Tempescul, Jean-Richard Eveillard, Norbert André, Frederic Morel, Isabelle Quintin-Roué, Christian Berthou

**Affiliations:** 1 Institut de Cancéro-hématologie, Département d'Hématologie, Hôpital Morvan, CHRU Brest, France; 2 Departement de Pneumologie, Hopital Cavale Blanche, Brest, France; 3 Laboratoire de Cytogénétique, Faculté de Médecine, Université de Bretagne Occidentale, Brest, France; 4 Laboratoire d'Anatomopathologie, Hôpital Morvan, CHRU Brest, France

## Abstract

**Background:**

Mantle cell lymphoma is a lymphoid entity characterized by adenopathy, blood and bone marrow involment which only recurrent mucosal localisation is the lymphomatoid polyposis. Few other mucosal infiltrations have been already reported.

**Results:**

We report here the first case of a unique tracheal localisation of mantle cell lymphoma at presentation of the disease. The presence of classical t(11;14)(q13;q32) confirmed the diagnosis of mantle cell lymphoma by eliminating MALT or cancer localisation.

**Conclusion:**

This case illustrates the necessity to ensure the diagnosis of mucosal lymphoma versus MCL since these diseases need different treatment regimens and prognoses.

## 

To the Editor,

We report here an unusual case of a tracheal localisation of mantle cell lymphoma (MCL). The patient was 75-years-old and hospitalized for dyspnoea, dysphonia and stridor, evolving from 3 months. No superficial tumoural syndrome was observed and the patient did not express B-symptoms. The CT-scan showed the presence of an endotracheal tumour of two centimetres under the glottis and two mediastinal centimetric lymph nodes. No other localisations were found. The bronchial endoscopy showed an obstructive vascularised tumour (Figure 1), and the stomach endoscopy was negative. The pneumologist took multiple biopsies and used both laser and endotracheal prothesis to treat the dyspnoea. The anatomo-pathologist identified a massive proliferation of medium to large cells with abundant and clear cytoplasm, round or oval nuclei. Mitosis were observed. Those cells were CD20+/bcl-2+ lymphoid cells with no lymphoepithelial lesions. Cells expressed the CD5+. Many lymphoid cells expressed Cyclin D1 (Monoclonal anti-mouse, clone SP4, Lab Vision). Some CD23+ dendritic cells were observed. CD138 and ALC stains were negative, excluding plasmacytoma and solid tumour. Fluorescence in situ hybridisation of tracheal tumour revealed the presence of a t(11;14)(q13;q32) translocation. We made the diagnosis of MCL. Furthermore, blood and bone marrow exams did not show any abnormal lymphoid B cells with cytological and molecular exams. The patient was treated with four courses of Vincristine-Adriamycin-Dexamethasone-Chloraminophene followed by four injections of Rituximab. We obtained a complete haematological, cytogenetical and isotopic remission. The patient is still alive and in complete remission, 4 years after the diagnosis.

**Figure 1:**
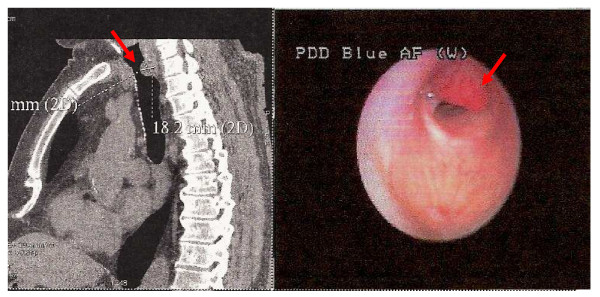
**The sagittal CT-scan showed the presence of an endotracheal tumour under the glottis (left panel)**. Presence of a vascularised tumor of the trachea visible by bronchial endoscopy (right panel).

Mantle cell lymphoma is a lymphoid entity defined by clinical, cytological, immunological, biochemical and cytogenetic criteria [1]. One particular entity of MCL, lymphomatoid polyposis, is characterised by the involvement of the gastrointestinal tract (30%), distinct from a mucosal associated lymphoid tissue (MALT) localisation [2,3]. The frequency of MALT in the trachea is very low; however, nasopharynx and Waldeyer's ring localisations of MCL mimicking MALT have been reported [4,5]. Dyspnoea was previously described in mediastinal involvement of MCL compressing the trachea [6]. Two cases have been already reported but there were relapse site or one of the multiple localisation of the MCL [7,8]. This case is different because of its unique localisation and the fact that it is the first evolution of the disease. Because tracheal involment is most seen in cancer and MALT lymphoma with different therapy and evolution, it is important to maximise the chance of an accurate diagnosis by correlating anatomo-pathologist and cytogenetic exams so as not to underestimate the incidence of atypical MCL in cancer/MALT localisation. This case illustrates the necessity to ensure the diagnosis of mucosal lymphoma versus MCL since these diseases have different treatment regimens and prognoses.

## Abbreviations

MALT: Mucosal Associated Lymphoid Tissue; MCL: Mantle Cell Lymphoma.

## Competing interests

The authors declare that they have no competing interests.

## Authors' contributions

JCI wrote the paper; AT JRE and CB collected the data and reviewed the paper; NA performed the endoscopic exam; FM did the cytogenetic exam and IQR performed the anatomopathologic exam. All authors read and approved the final manuscript.
